# Enhancing the management of anorexia of ageing to counteract malnutrition: are physical activity guidelines optimal?

**DOI:** 10.1007/s40520-022-02317-3

**Published:** 2023-01-20

**Authors:** Daniel R. Crabtree, Natalie J. Cox, Stephen E. R. Lim, Adrian Holliday

**Affiliations:** 1 Institute of Health Research and Innovation, Division of Biomedical Sciences, University of the Highlands and Islands, Inverness, UK; 2 Faculty of Medicine, Academic Geriatric Medicine, University of Southampton, Tremona Road, Southampton, UK; 3 NIHR Southampton Biomedical Research Centre, University of Southampton and University Hospital Southampton NHS Foundation Trust, Tremona Road, Southampton, UK; 4 NIHR Applied Research Collaboration Wessex, Southampton, UK; 5 University Hospital Southampton NHS Foundation Trust, Tremona Road, Southampton, UK; 6 Human Nutrition Research Centre, Population Health Sciences Institute, Newcastle University, Newcastle Upon Tyne, UK

**Keywords:** Appetite, Physical activity, Ageing, Anorexia of ageing, Malnutrition

## Introduction

Poor appetite in later life—termed “anorexia of ageing”—is acknowledged as a key determinant of age-related malnutrition. While physical activity (PA) is often recommended for increasing drive to eat, these recommendations are not well-evidenced in the older population. In this opinion piece we outline limitations to physical activity recommendations in anorexic older adults. We then discuss current evidence for the relationship between physical activity and appetite amongst younger adults and postulate how this relationship may change in later life, with implications regarding future recommendations and research.

## Malnutrition and anorexia in later life

The older adult population is particularly heterogeneous. Somewhat paradoxically those aged 65–75 years in the United Kingdom (UK) are at greater risk of both excess body mass and low body mass, compared with young- and mid-life adults [1]. However, it is low body mass and a loss of mass, rather than excess body mass or gains in mass, that pose the greatest risk of mortality in older adults [2, 3]. As such, malnutrition—in this context referring to a lack of intake of nutritional requirements leading to loss of body mass and function—is a major healthcare challenge in older people, contributing to negative health outcomes, including sarcopenia and frailty, and adversely affecting independence and quality of life, to large healthcare cost [4]. It is estimated that around 27% of community dwelling older adults and up to 50% of those in healthcare settings are at risk of malnutrition [5]. The management of malnutrition in older adults, such as promotion of higher protein intake, has progressed over recent years. However, knowledge regarding optimal approaches to prevent or delay its onset and to slow its progression are less well established [4]. The aetiology of malnutrition in older people is multifactorial (see Volkert et al. [6] for a comprehensive overview) but one key determinant is loss of appetite, which is highly prevalent across settings, affecting 20% of community dwelling older adults and rising to over 40% in hospitalised populations [4, 7]. Anorexia can be due to effects of comorbidity or medications but can also be a consequence of the ageing process itself acting to reduce overall energy intake (EI) and negatively alter dietary pattern [7]. Stimulating appetite and thus EI is an important potential strategy to prevent onset or reduce progression of malnutrition.

There are few well-evidenced strategies to stimulate appetite in anorexic older adults [7]. Popular belief, alongside guidance from ageing charities and health agencies such as the National Health Service (NHS) in the UK, advocate increasing PA as a method to improve appetite in later life [8]. Current UK PA recommendations for those over 65 years include daily activity to accumulate 150 min of moderate intensity activity (such as brisk walking) a week and resistance activity on at least 2 days a week [9]. Higher levels of habitual PA have been proposed to increase the drive to eat and improve matching of energy intake with energy expenditure. However, evidence for this relationship stems from observations on adults aged under 65 years, while the nature of this association in older populations is largely unexplored.

Inadequate PA is a ubiquitous problem. A study which included data from 358 surveys across 168 countries (*n* = 1.9 million) showed a global prevalence of insufficient PA of 23.3% [10]. Engagement in PA differs between older and mid-life adults, with a trend towards reduction in PA with advancing years. A survey conducted in England in 2016 showed that more than 60% of men and women aged between 45 and 54 years met the recommended aerobic activity guidelines [11]. In contrast, only 35% of men and 24% of women aged over 75 years met these guidelines [11].

Given the differences in PA between older and mid-life adults, it is questionable whether current guidance to follow recommended PA levels as a means to stimulate appetite for older adults is informed by sufficient strength of evidence. It is therefore important to interrogate potential differences in the relationship between habitual PA and appetite control for mid and later adult life to guide future research. This would provide evidenced PA recommendations with a view to stimulate appetite in older adults with anorexia and at risk of malnutrition.

## The relationship between physical activity and appetite control: the J-shaped curve theory

It has been proposed that being physically active enhances the sensitivity of appetite control and is associated with improved coupling of EI with energy expenditure (EE). Seminal work by Mayer et al*.* [12] examined the relationship between caloric intake and habitual PA in a group of 213 mill workers, whose daily activities were categorized and ranged from sedentary to very hard physical labour. Mayer et al. [12] found that those workers with high PA increased daily EI to match EE, while at lower PA energy coupling appeared dysregulated and instead of observing a proportional decrease in daily EI to accompany a reduction in EE, EI was found to increase.

This seemingly J-shaped relationship between habitual PA and EI has been substantiated and developed since the initial findings of Mayer et al*.* [12]. More recent studies, predominantly conducted with adults aged < 65 years, have found that active individuals reported elevated feelings of hunger and greater EI in comparison with inactive individuals. Furthermore, it has been demonstrated that those who regularly engage in moderate-to-vigorous PA reduce EI after high energy vs low energy preloads, while those who are less active do not adjust subsequent EI to compensate for preload energy content. This indicates a tendency for more active people to adjust feeding to achieve an acute coupling of energy intake with energy needs. Thus, as individuals progress along the continuum of habitual PA towards higher levels of activity, the drive to eat increases (to offset the increase in physical activity energy expenditure (PAEE)) and postprandial satiety signalling strengthens. In contrast, those with lower levels of PA are perhaps more inclined to engage in non-homeostatic feeding behaviours, attributed to excess adiposity weakening satiety signalling [13]. In 2011, Professor John Blundell insightfully proposed the concept of appetite control zones in relation to the J-shaped curve theory. Blundell [14] purported that inactive individuals occupy a “non-regulated zone” of appetite control, in which satiety signalling is blunted, causing overeating and consequential weight gain. Conversely, a sustained increase in PA improves physiological regulation of energy balance, shifting those who are more active into what Blundell [14] termed the “regulated zone” of appetite control. Here, tonic appetite and EI is driven predominantly by the energy demand of resting metabolic rate (RMR) and PAEE, with active people demonstrating largely effective energy coupling.

The J-shaped model was most recently revised by Beaulieu et al*.* [15] to include the relationship between habitual PA and food reward. Beaulieu et al. [15] observed an association between low PA and greater liking and wanting for high-fat foods, while higher PA appeared to be associated with a reduced wanting for high-fat foods and a greater liking for low-fat foods. Therefore, although increasing PA may concomitantly increase the orexigenic drive to eat, PA-induced changes to the hedonic processing of food cues could stimulate healthier food choices, thereby promoting more effective weight management.

## Physical activity and appetite control in later life: a distorted J-shaped relationship?

**Fig. 1:**
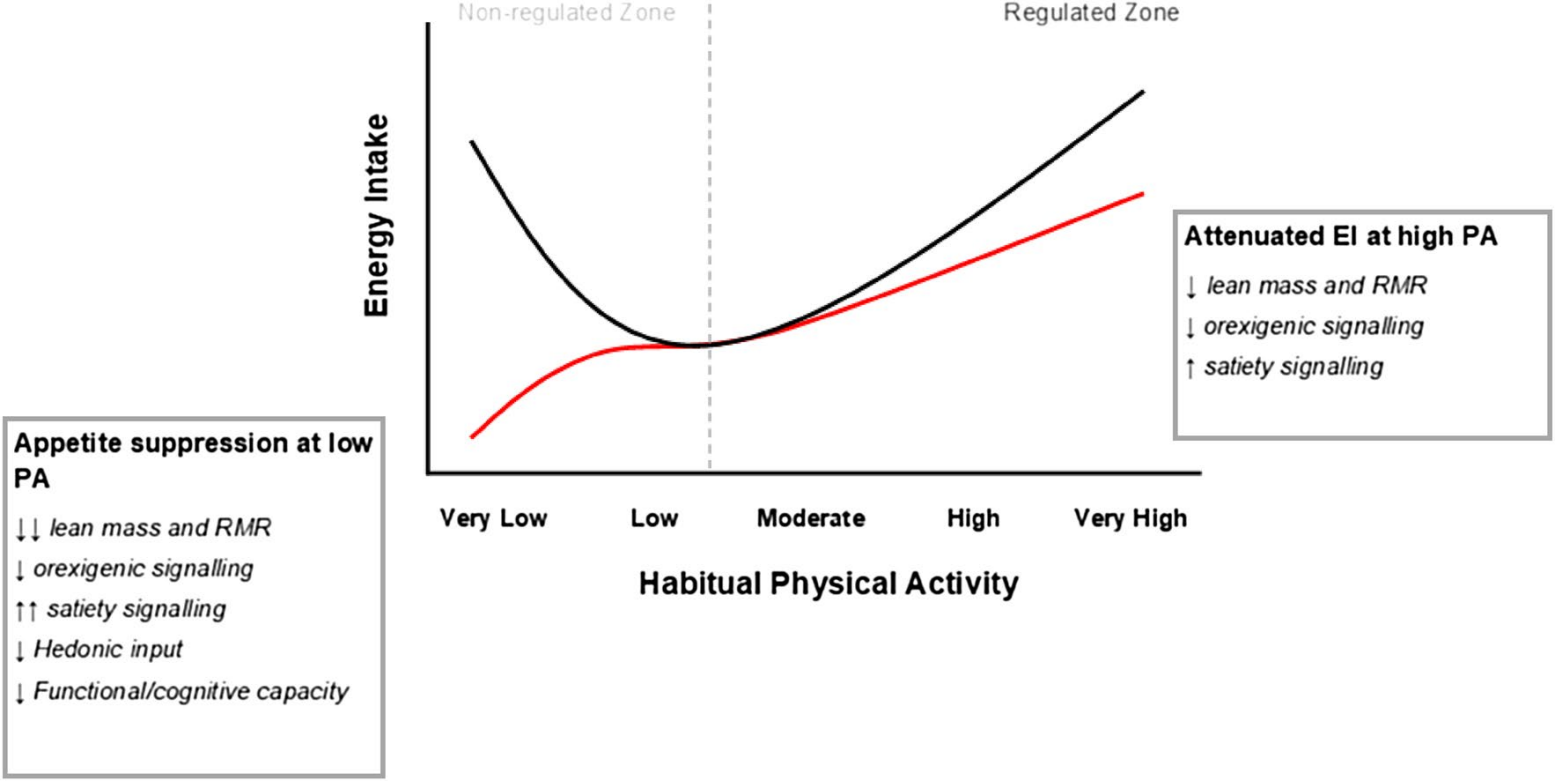
The well-established J-shaped relationship between habitual PA and EI in younger adults (black line), and the proposed distorted J-shaped relationship between habitual PA and EI in older adults (red line)

The J-shaped curve may not represent the association between habitual PA and EI in older adults. The impact of PA on appetite control has predominantly been studied in overweight and obese younger adults, and our understanding of how PA influences appetite and energy coupling within the context of ageing is limited. It should perhaps not be assumed that the relationship between PA and appetite control that has been demonstrated in young adults also applies to older adults. We postulate, with theoretical basis, a distorted J-shaped relationship (Fig. 1) may be more representative. In the regulated zone, we propose a shallower curve, representing a somewhat attenuated increase in EI with increasing PA. Due to sarcopenic change and age-related anabolic resistance, physically active older adults likely possess less lean mass than equally active younger adults. Consequently, resting metabolic rate (the greatest determinant of which is fat-free mass), total energy expenditure, and hence drive to eat are expected to be lower. This may be exacerbated by inhibited orexigenic signalling via dysregulation of the ghrelin axis. Further, evidence points to augmented hormonal anorexigenic signalling with ageing, providing a mechanism by which older adults consume smaller intakes than younger adults, even when drive to eat is high. A recent meta-analysis by Johnson and colleagues [16] highlights greater fasted and postprandial circulating concentrations of the anorexigenic hormones leptin, insulin and cholecystokinin (CCK), and greater postprandial concentrations of peptide tyrosine tyrosine (PYY) in older adults, compared with younger adults. Such hormonal responses are associated with reduced appetite and EI.

At the other end of the PA spectrum, we hypothesise a non-regulated zone, but one in which EI is decreased at very low activity levels in older adults (Fig. 1). This is in contrast to the high intakes seen with low activity in young and mid-life adults. There are several mechanisms by which we propose a differing response with ageing. Firstly, given that activity levels decline with age, a “low activity” older adult will likely be less active than a “low activity” younger adult. Very low activity, coupled with sarcopenia, likely renders inactive older adults with very low lean mass. Consequently, both RMR and PAEE will be extremely low, severely reducing the drive to eat. This again may be exacerbated by inhibited orexigenic signalling via dysregulation of the ghrelin axis. The non-regulated zone of the J-shaped curve is attributed, in part at least, to dysregulation in hormonal satiety signalling and increased hedonic drive associated with the increased adiposity of those of low activity level [13]; high adiposity “over-rides” the energy coupling mechanisms which prevail at higher activity and lower adiposity levels. However, the age-related augmentation of hormonal satiety signalling is likely to mitigate any adiposity-related inhibition of hormonal satiety signalling. Further, hedonic inputs reduce with age due to weakening senses of taste and smell [4]. Add to this, possible declines in functional and cognitive capacity that can accompany ageing and frailty, which can limit capacity to access and prepare food [4] (essentially offering some degree of immunity to the Western obesogenic environment), and there are numerous potential reasons for malnutrition in low activity older adults.

Such a relationship between low PA and low EI would perpetuate a vicious cycle of malnutrition and frailty (see Fig S1 in Supplementary Material). Many characteristics of low activity are also closely related to malnutrition (low mass, low functional capacity). It is easy to see how a sudden reduction in PA (perhaps due to illness or injury) and consequent acceleration in loss of lean mass could elicit a reduction in drive to eat and EI. This will further exacerbate lean mass loss, potentially impairing functional capacity, which in turn reduces PA. Hence, this cycle can accelerate sharply, with severe reciprocal reductions in both PA and EI, and potentially catastrophic consequences for health and wellbeing.

## So, what next? Future research and potential implications

Studies are required to substantiate our hypothesised distorted J-shaped relationship between PA and EI in older adults. Cross-sectional data will be informative and is perhaps the first avenue to pursue for future research. Of particular importance, however, is understanding the impact of increasing PA on appetite and EI, and consequently on the risk of malnutrition. Our model suggests that moving undernourished older adults from “very low” or “low” activity levels to a “moderate” activity level might provide substantial benefits to appetite and EI.

As such, we propose that PA should form a fundamental component of treatment for anorexia of ageing. However, the lack of evidence-base to support current generic PA recommendations provided by health agencies with regards the effect of PA on appetite and EI in older adults limits PAs utility as a treatment option, rendering it potentially suboptimal for treating anorexia of ageing.

Studies are needed to inform and optimise the amount and nature of PA recommended for those with anorexia of ageing, with a consideration of the likely acute and chronic impact on appetite and energy balance. Firstly, fully elucidating the relationship between PA and EI in older adults may highlight a PA “threshold”, at which those below suffer a marked reduction in appetite and EI. If this is the case, supporting older adults to exceed this PA “threshold” should be of paramount importance when treating anorexia of ageing. Secondly, the benefits of resistance exercise for older adults with regards maintaining lean mass and physical capacity are well established. Maintaining, or even increasing lean mass, is also important for maintaining drive to eat, through a maintenance or increase in RMR. In addition, while high-intensity exercise typically induces a transient suppression of appetite, resistance exercise has been shown not to negatively impact short-term EI in older adults [17]. Therefore, there may be an argument for resistance exercise to be a more prominent component of PA recommendations for older adults with low appetite; it may be beneficial, from an appetite and EI perspective, to exceed the current guidelines of completing resistance exercise on two days per week.

Through further research and a greater understanding of the relationship between habitual PA and eating behaviour, we can progress to more effective, evidence-based practise in the management of anorexia of ageing.

## Supplementary Information

Below is the link to the electronic supplementary material.

Supplementary file1 (DOCX 72 KB)
