# Supplementary material for: Enhancing the management of anorexia of ageing to counteract malnutrition: are physical activity guidelines optimal?
Source: Aging Clin Exp Res. 2023 Jan 20;35(2):427–31. doi: 10.1007/s40520-022-02317-3 (PMC9894952; doi:10.1007/s40520-022-02317-3)
Supplement: Supplementary file 1 — Supplementary file1 (DOCX 72 KB) [file 40520_2022_2317_MOESM1_ESM.docx]

**Supplement 1 – Fig S1**


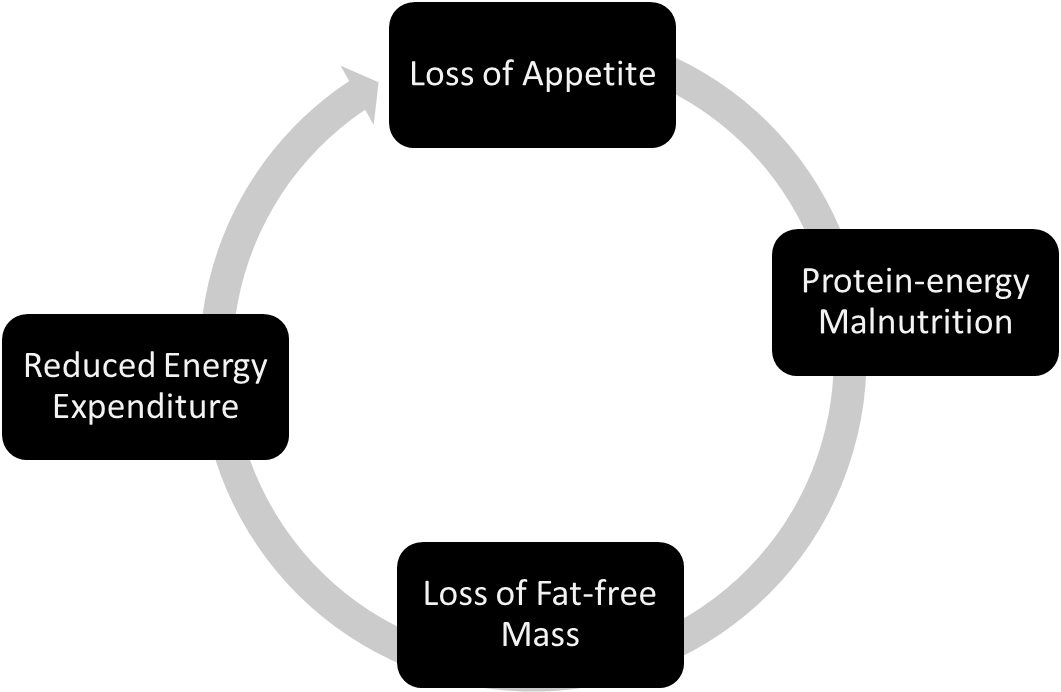


**Vicious Cycle of Anorexia, Malnutrition,**

**and Frailty**

↓ Food intake

↓ MPS

↓ RMR

Illness/Injury

↓PA

Immobilisation

Reduced Physical Activity

**Figure S1**. The vicious cycle of anorexia, malnutrition and frailty. A loss of appetite contributes to a cycle of reduced protein and energy intake, loss of fat-free mass and consequent reduction in resting metabolic rate (RMR). As energy expenditure, of which RMR is the greatest contributor, is the primary driver of appetite, this further reduces the drive to eat, perpetuating the cycle. The cycle is exacerbated by inactivity and accelerated by illness and injury which lead to immobilisation and a consequent augmented rate of fat-free mass loss. PA = physical activity; MPS = muscle protein synthesis.
